# A Novel Lightweight Framework for Non-Contact Broiler Face Identification in Intensive Farming

**DOI:** 10.3390/s25134051

**Published:** 2025-06-29

**Authors:** Bin Gao, Yongmin Guo, Pengshen Zheng, Kaisi Yang, Changxi Chen

**Affiliations:** 1 Key Laboratory of Smart Breeding (Co-Construction by Ministry and Province), Ministry of Agriculture and Rural Affairs, Tianjin 300384, China; bingao_1594@163.com (B.G.); guoyongmin8@163.com (Y.G.); 163109306@stud.tjut.edu.cn (K.Y.); 2 College of Computer and Information Engineering, Tianjin Agricultural University, Tianjin 300384, China; zhengpengshen_9206@163.com

**Keywords:** lightweight convolutional neural network, multi-scale feature fusion, broiler face recognition, precision livestock farming

## Abstract

Efficient individual identification is essential for advancing precision broiler farming. In this study, we propose YOLO-IFSC, a high-precision and lightweight face recognition framework specifically designed for dense broiler farming environments. Building on the YOLOv11n architecture, the proposed model integrates four key modules to overcome the limitations of traditional methods and recent CNN-based approaches. The Inception-F module employs a dynamic multi-branch design to enhance multi-scale feature extraction, while the C2f-Faster module leverages partial convolution to reduce computational redundancy and parameter count. Furthermore, the SPPELANF module reinforces cross-layer spatial feature aggregation to alleviate the adverse effects of occlusion, and the CBAM module introduces a dual-domain attention mechanism to emphasize critical facial regions. Experimental evaluations on a self-constructed dataset demonstrate that YOLO-IFSC achieves a mAP@0.5 of 91.5%, alongside a 40.8% reduction in parameters and a 24.2% reduction in FLOPs compared to the baseline, with a consistent real-time inference speed of 36.6 FPS. The proposed framework offers a cost-effective, non-contact alternative for broiler face recognition, significantly advancing individual tracking and welfare monitoring in precision farming.

## 1. Introduction

The broiler chicken industry plays a critical role in driving economic growth, ensuring market supply, and increasing farmers’ incomes [1,2]. With the rapid development of broiler production, the implementation of precision farming management has become increasingly important [3]. In modern broiler rearing and breeding, comprehensive collection of individual data facilitates the formulation of scientific breeding strategies, disease prevention, and the mitigation of potential risks in farms [4,5]. Accurate recognition of individual broilers is a prerequisite for obtaining detailed individual information. By employing precise individual recognition technology, producers can monitor the health and welfare of each broiler in real time, thereby devising personalized management plans and enhancing overall farming welfare [6]. Furthermore, individual broiler recognition plays an irreplaceable role in behavioral studies and full-process product traceability [7,8]. In recent years, with the widespread adoption of sustainable intensive farming models, the demand for accurate individual recognition of every broiler has become increasingly urgent.

Currently, radio frequency identification (RFID) technology has become one of the most widely used methods for individual identification in broilers. Gao’s team developed an intelligent weighing system based on RFID to accurately capture individual broiler weights [9]. Similarly, van der Sluis and colleagues constructed a tracking system using RFID tags that enables real-time monitoring of broilers’ behaviors throughout their lifecycle [10]. In addition, Collet employed RFID technology to collect positional data of individual broilers, thereby achieving precise quantification of free-range behavior [11]. However, despite its widespread application in broiler identification, RFID technology faces numerous challenges in large-scale farms. Primarily, RFID systems are susceptible to interference from environmental factors—especially nearby electronic devices and metallic components—which can reduce chip recognition accuracy [12]. Moreover, the high cost of sensor deployment and maintenance, along with stringent requirements for power consumption and device performance stability, further limit its application. More importantly, the use of RFID technology may have adverse impacts on broiler welfare [13]. Compared with traditional methods, contactless identification approaches based on broiler facial features offer the advantages of non-invasiveness, higher stability, and improved recognition accuracy. Consequently, the advancement of facial recognition techniques for broiler chickens holds great promise for facilitating precision poultry farming, enhancing animal welfare, and meeting the growing demands for intelligent and efficient management in modern agricultural systems.

With continuous advances in computer vision technology, an increasing number of researchers have adopted non-contact methods to conduct studies on broiler target detection, pose estimation, as well as growth and health monitoring [14,15]. For example, Ma et al. constructed an advanced chicken face detection network based on Generative Adversarial Networks (GANs) and Masked Autoencoders (MAEs), enhancing small-object features through adversarial generation and self-encoding mechanisms [16]; Yin et al. integrated multi-object tracking with single-shot detection to enable long-term behavioral monitoring and individual tracking of broilers [17]; Yang et al. utilized deep learning models to analyze movement trajectories within broiler flocks, achieving accurate detection and classification of motion states, which supports activity assessment and early health warning [18]; Narisi et al. estimated the feeding time of individual broilers using convolutional neural networks (CNNs) combined with image processing techniques, validating its application potential in optimizing feed efficiency [19]. However, research on broiler face recognition remains in its infancy and lags significantly behind the facial recognition technologies developed for larger livestock such as pigs and cattle. For instance, Hansen’s team developed a pig face recognition method based on convolutional neural networks (CNNs) that achieved 96.7% accuracy in farm environments [20], while Weng and colleagues proposed the TB-CNN model, which attained 99.85% accuracy in multi-angle cattle face recognition [21]. In contrast, broiler face recognition has yet to achieve similar breakthroughs, and no dedicated studies on this topic have been reported. This technological gap mainly stems from three specific challenges: first, the relatively small facial area and subtle features of broilers demand higher recognition accuracy [16]; second, mutual occlusion among broilers further complicates facial feature extraction [22]; and third, recognition errors in dynamic multi-target scenarios tend to escalate as group size increases [23]. Moreover, due to the limited computational resources of embedded systems, CNN-based deep learning algorithms must optimize memory management, reduce weight parameters, and lower computational costs to meet the requirements of embedded deployment [24,25].

Given the urgent need for lightweight, high-precision solutions, YOLO-based architectures emerge as a promising candidate due to their inherent advantages in speed-accuracy trade-offs and embedded deployment feasibility [26]. Modern YOLO variants demonstrate exceptional deployment efficiency, achieving ultra-high frame rates through one-stage inference on conventional hardware while maintaining detection performance, making them particularly suitable for real-time monitoring requirements [27]. For instance, the YOLO Granada framework, derived from YOLOv5, reduces parameter counts and FLOPs to approximately 55% of the original model through network pruning and lightweight architectural design, concurrently enhancing inference speed by 17% [28]. The LSR YOLO model optimized for sheep face detection employs ShuffleNetv2/Ghost modules to replace standard convolutions, achieving a compact model size of ~9.5 MB specifically tailored for edge devices [29]. On resource-constrained platforms like Jetson Nano, Tiny YOLOv3 achieves ~9 FPS (≈110 ms/frame) [30], while the Ag YOLO optimized for low-power NCS2 accelerators attains 36.5 FPS with a 12-fold parameter reduction [31]. The latest YOLOv11 architecture incorporates cross-stage spatial attention modules and network reconfiguration designs, effectively enhancing multi-scale feature representation capabilities without compromising real-time performance through improved contextual feature focusing [32]. Multiple optimization strategies including network pruning, lightweight module substitution, and attention mechanism enhancement have been systematically validated to improve deployment feasibility in embedded systems while maintaining computational efficiency.

To address the array of challenges in individual broiler identification, this study proposes a broiler face recognition model named YOLO-IFSC. The model is specifically optimized based on YOLOv11n to tackle the aforementioned issues. First, by refining the network structure, YOLO-IFSC significantly improves recognition accuracy for small targets and subtle features, effectively mitigating the high-precision demands imposed by the small facial area. Second, the model enhances its robustness against occlusion, effectively reducing the interference in facial feature extraction caused by occluded regions. Additionally, YOLO-IFSC employs multi-scale feature fusion techniques to resolve multi-target recognition challenges in group environments, and its lightweight design reduces computational cost and memory usage, thereby meeting the practical requirements of embedded devices. The main improvements to YOLOv11n are as follows:

Inception-F Module Replacement: A multi-branch dynamic fusion module is introduced, which optimizes multi-scale feature extraction through parallel pathways and dynamic weighting mechanisms [33]. This enhancement increases the model’s adaptability to features at various granularities while effectively reducing both the computational load and parameter count in the broiler face recognition task, thereby improving overall computational efficiency.

C2f-Faster Module Replacement: An efficient feature compression module based on partial convolution is adopted [34]. By leveraging selective channel computation to reduce redundancy, this module strikes an excellent balance between computational cost and feature representation capability.

SPPELANF Module Replacement: A multi-scale pyramid fusion module is introduced, which combines cross-level feature integration with dynamic pooling strategies to enhance the model’s ability to perceive complex spatial relationships [35].

CBAM Module Replacement: A dual-domain attention-guided module is utilized, which adaptively weights both the channel and spatial dimensions, effectively enhancing the feature response in key regions [36].

## 2. Materials and Methods

### 2.1. Image Acquisition

**Figure 1:**
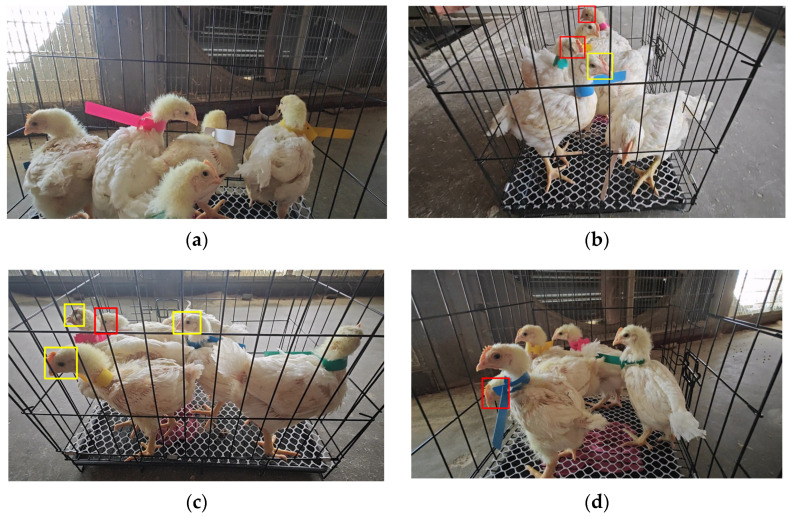
Panels (**a**–**d**) illustrate images captured from various angles and distances. In panel (**a**), no facial occlusion is present, whereas panels (**b**–**d**) show natural occlusions among broilers indicated by red boxes and occlusions from the cage environment indicated by yellow boxes.

All data for this study were collected from Smart Poultry Farm in Pingyuan, Shandong Province, China, comprising facial images of 24 WOD168 white-feather broilers (aged 16–22 days) reared in cages. To achieve a cage-rearing stocking density of 30 kg of live weight per square meter [37], we designed a standardized data-collection cage measuring 50 × 35 × 41 cm to accommodate 4–6 broilers per cage. As shown in Figure 1, videos were captured from multiple angles using an iPhone 13 smartphone (Apple Inc., Cupertino, CA, USA. resolution: 3840 × 2160, 30 fps) to ensure natural interactions and occlusions among the broilers. After manually inspecting and discarding blurred images, a total of 3541 raw images were obtained.

### 2.2. Construction of the Broiler Face Dataset

To develop a broiler face recognition model capable of handling complex scenarios, this study optimized dataset quality and expanded its scale through redundancy filtering and data augmentation.

#### 2.2.1. Redundancy Filtering

Initially, the Structural Similarity Index Measure (SSIM, with a threshold set at 0.65) was applied to consecutive frames to eliminate highly repetitive static scenes. Following this preprocessing, 839 valid images were retained from the original 3541 images. Each image contains between 2 and 6 broiler faces, forming a densely distributed dataset.

#### 2.2.2. Data Annotation and Data Augmentation

To ensure that the test set comprehensively covers all 24 broiler individuals under the varied viewing angles, distances, and occlusion scenarios illustrated in Figure 1, we first performed stratified sampling on the entire set of 839 raw images, selecting 108 images to form the test set. From the remaining 731 images (after removing the test set), we again applied the same stratification criteria to select 216 images for the validation set, thereby securing stable and generalizable performance metrics during hyperparameter tuning. The remaining 515 images were designated as the training set.

Using LabelImg, we manually annotated broiler faces in the training, validation, and test sets, distinguishing individual identities by the colored neck tags shown in Figure 1. The annotation statistics for the raw images are as follows: the test set (108 images) contains 416 broiler face instances; the validation set (216 images) contains 852 instances; and the original training set (515 images) contains 2164 instances.

**Figure 2:**
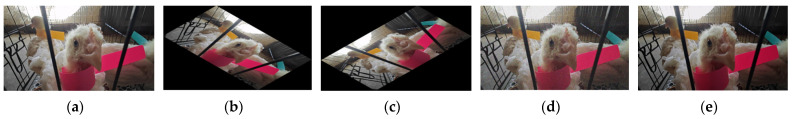
Panel (**a**) presents original data; panel (**b**) shows image rotated 45° clockwise; panel (**c**) displays image rotated 45° counterclockwise; panel (**d**) demonstrates addition of 0.5–3% salt-and-pepper noise; and panel (**e**) illustrates introduction of an illumination gradient.

**Figure 3:**
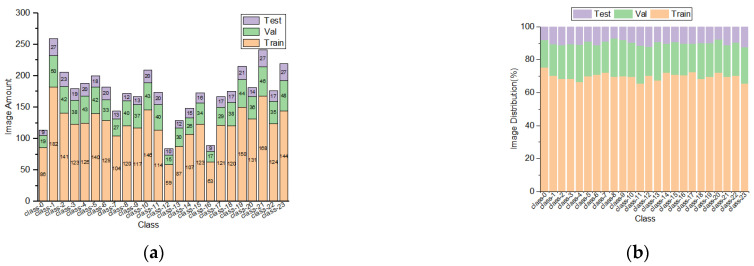
(**a**) Number of annotation instances for each of the 24 broilers in training, validation, and test sets; (**b**) percentage distribution of annotation instances for each of the 24 broilers across training, validation, and test sets.

To address the dynamic challenges of broiler face recognition in real-world conditions, we designed three data augmentation strategies to enhance model robustness, as depicted in Figure 2. First, we applied random rotations between −45° and +45° to simulate natural head movements, improving pose invariance [38]. Second, salt-and-pepper noise at densities of 0.5–3% was added to imitate dust accumulation on the lens and sensor degradation, thereby enhancing noise resilience [39]. Finally, random gamma correction (γ ∈ [0.5, 1.5]) combined with HSV brightness perturbations (±20%) simulated illumination changes across different time periods [40]. Through these augmentation techniques, 241 derived images were generated from the original training set, and their annotations were updated synchronously—rotation operations required geometric coordinate transformations to generate new bounding boxes, while noise and brightness adjustments reused the original annotations—resulting in an additional 761 annotated instances. Consequently, the training set expanded to 756 images (original 515 + augmented 241), containing 2925 total face annotations. Overall, the three subsets (test 108, validation 216, training 756) encompass 4193 annotated instances, as detailed in Table 1 and Figure 3.

### 2.3. Construction of the Broiler Face Recognition Model

#### 2.3.1. Construction of the YOLO-IFSC Model

**Figure 4:**
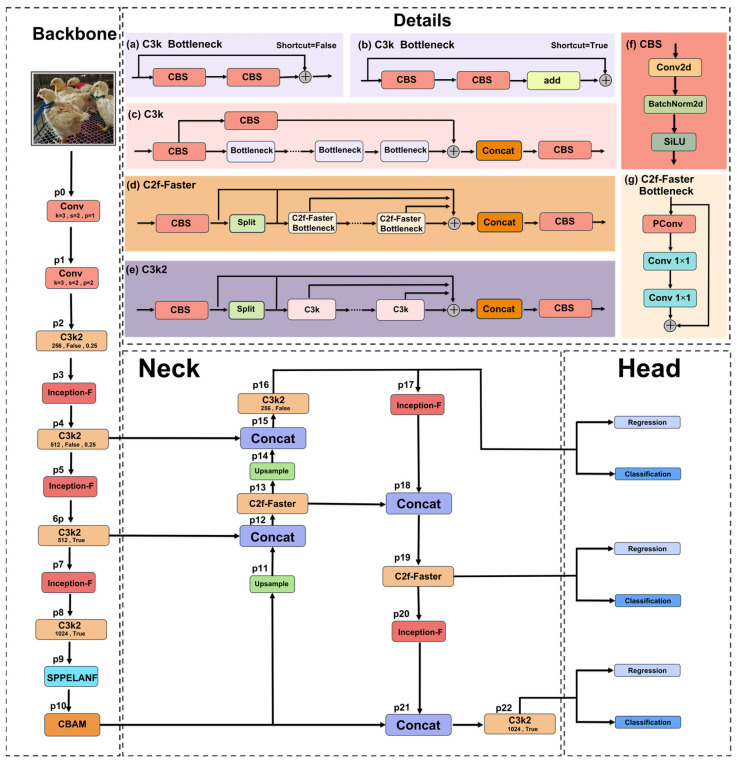
YOLO-IFSC architecture.

In this study, we innovatively improved the YOLOv11n model by proposing the YOLO-IFSC model, whose architecture is shown in Figure 4. To enhance small object detection efficiency and reduce computational cost, the Inception-F module is applied to the p3, p5, and p7 layers in the backbone network, as well as the p17 and p20 layers in the neck network. The C2f-Faster module is introduced to replace the p13 and p19 layers in the neck network, improving feature extraction efficiency and reducing computational complexity. Furthermore, the SPPF module is substituted with the SPPELANF module (at p9), which addresses occlusion issues in broiler face recognition by utilizing multi-scale feature fusion, thus improving recognition accuracy. To further refine the model’s performance, the C2PSA module is replaced by the CBAM module (at p10), which enhances the focus on key facial features. These modifications collectively improve both the efficiency and accuracy of broiler face recognition, making the YOLO-IFSC model suitable for real-time deployment on edge devices.

#### 2.3.2. YOLOv11 Model

Starting with YOLOv5, the YOLO series has gradually transitioned to the PyTorch framework, establishing itself as one of the leading technologies in object detection. With the release of YOLOv8, the YOLO framework has achieved significant improvements in both accuracy and speed [41]. Compared to YOLOv8, YOLOv11 incorporates several optimizations on top of the YOLOv8 base model. Firstly, the backbone utilizes the C3K2 (c3k = False/True) module in conjunction with a cascade of the SPPF and C2PSA modules, further enhancing feature extraction and information fusion capabilities. The neck section achieves multi-scale information integration through up-sampling and feature concatenation, and it replaces the original C2f module with a more efficient C3K2 (c3k = False/True) module to further optimize feature fusion. Additionally, the head consists of multiple layers of C3K2 and CBS modules, and it generates detection predictions through a series of convolutional layers, thus completing the tasks of object localization and classification [32].

#### 2.3.3. Inception-F Model

Inspired by the concepts of the Inception series network modules [33] and the depthwise separable convolution in Xception [42], this study proposes an identification module—Inception-F—specifically optimized for the feature characteristics of broiler face data.

**Figure 5:**
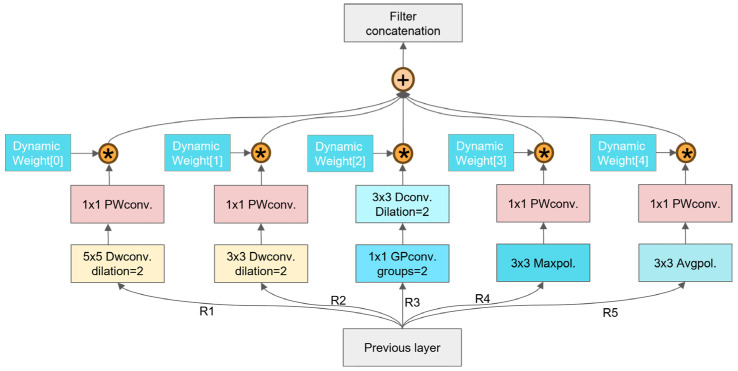
Inception-F module structure diagram.

As shown in Figure 5, the R1 and R2 paths combine depthwise separable convolution (with a dilation rate of 2) and pointwise convolution to efficiently extract local fine details from broiler face data [43], while dilated convolution expands the receptive field to enhance the capture of complex backgrounds and small targets. Depthwise separable convolution decomposes standard convolution into channelwise convolution and pointwise convolution, effectively reducing computational load and parameter count, thereby improving efficiency [44]. In addition, pointwise convolution further refines feature representation through streamlined channel fusion, enhancing discriminative capability. Notably, the R2 path employs a 5 × 5 convolution to compensate for R1’s limitations in processing larger targets; the larger convolution kernel integrates more contextual information and strengthens multi-scale target recognition [45]. Given that the extended convolutional range in the R2 path may introduce additional computational burden and potential information loss, the R3 path incorporates a 1 × 1 grouped convolution to achieve efficient and fine-grained channel information fusion, thereby reducing computational complexity and effectively mitigating the limitations in channel feature integration observed in R1 and R2. Finally, the R4 and R5 paths introduce max pooling and average pooling operations [46], respectively, to further optimize spatial feature representation while reducing computational load and memory usage.

Moreover, this design incorporates a dynamic weighting mechanism to adaptively adjust the contribution of each path during feature extraction, thereby optimizing feature fusion and enhancing the model’s representational capacity. To further explain the dynamic weight fusion process, let the outputs of the five paths be denoted as *x*_1_, *x*_2_, *x*_3_, *x*_4_, and *x*_5_, with corresponding dynamic weights *w*_1_*′*, *w*_2_*′*, *w*_3_*′*, *w*_4_*′*, and *w*_5_*′* ∈ ℝ. These weights are normalized using the Softmax function to ensure a probabilistic interpretation:

(1) $$wi=\frac{\exp(w_{i}^{\prime })}{\sum_{j}^{5}\exp(w_{i}^{\prime })},\forall i\in \{1,\ldots ,5\}$$

The normalized weights satisfy $\sum_{i}^{5}w_{i}=1$ and $w_{i}\in [0,1]$.

The fused feature $y\in R^{C\times H\times W}$ is computed as follows:

(2) $$y=\sum_{i=1}^{5}wi⊙{\hat{x}}_{i}$$

In the equation, ⊙ denotes element-wise multiplication.

The proposed Inception-F module, with its multi-path design and dynamic weight fusion mechanism, effectively integrates multiple feature extraction strategies. It alleviates challenges in broiler face recognition due to small targets, occlusions, and multi-target scenarios, significantly enhancing the extraction of fine-grained and multi-scale features, while also demonstrating clear advantages in computational efficiency—making it suitable for practical applications on embedded systems.

#### 2.3.4. C2f-Faster Module

In broiler face recognition tasks, the small size and subtle features of the broiler face region pose efficiency bottlenecks for traditional convolution in local information extraction. To enhance feature extraction efficiency and optimize computational performance, this study proposes the C2f-Faster module (as shown in Figure 4d). In this module, the Bottleneck of C2f-Faster (illustrated in Figure 4g) is an optimized version of the Bottleneck in the C3k structure (shown in Figure 4a). The specific optimization strategies include replacing the initial standard convolution with partial convolution (PConv) to reduce redundant computations, and decomposing the subsequent standard convolution into two 1 × 1 convolutions to bolster feature fusion capabilities [34]. Furthermore, the optimized Bottleneck is incorporated into the C2f structure to form the C2f-Faster module, thereby enhancing the model’s feature representation and computational efficiency [47]. The introduction of PConv enables the model to selectively focus on key channel features, so that even if parts of the target are occluded, information from unobstructed regions can still be utilized. Additionally, the use of 1 × 1 convolutions enhances inter-channel information exchange, improving the model’s ability to represent edge, texture, and shape features, and thus enhancing target recognition performance under occlusion.

**Figure 6:**
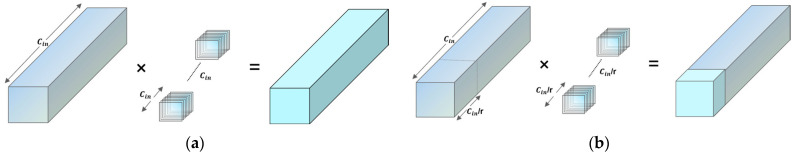
(**a**) Standard convolution, (**b**) PConv convolution.

From a computational complexity standpoint, Figure 6 compares the processes of standard convolution and PConv. Standard convolution requires computations across all channels, resulting in high FLOPs (floating-point operations per second). In contrast, PConv applies convolution selectively to a subset of the input channels, effectively reducing computational load and memory access. FLOPs can be expressed as:

(3) $${FLOPs}_{PConv}=K_{h}\times K_{w}\times C_{in}\times C_{out}\times H\times W\times P$$

where P represents the proportion of the channel subset, typically set to 1/4, so that the computational overhead of PConv is significantly lower than that of standard convolution.

Overall, the C2f-Faster module, while maintaining high computational efficiency, enhances contextual awareness, rendering the model more robust in broiler face recognition tasks. It is capable of sustaining high recognition accuracy and computational efficiency even under conditions of occlusion, multi-target interference, and resource constraints.

#### 2.3.5. SPPELANF Module

Spatial Pyramid Pooling (SPP) demonstrates strong robustness and accuracy in feature extraction, but its high computational complexity limits the feasibility of real-time applications [48]. To address this issue, Spatial Pyramid Pooling Acceleration (SPPF) employs sequential, same-scale max pooling operations to improve the efficiency of multi-scale feature extraction. However, its serial design limits the integration of global context information, which can lead to inconsistent feature representation in complex broiler face occlusion scenarios.

To overcome this challenge, we propose the SPPELANF module, which combines the parallel pooling architecture of SPP with the design principles of Efficient Layer Aggregation Networks (ELANs) [35]. This design enables the parallel capture and hierarchical aggregation of multi-scale features, enhancing the retention of local details while improving global semantic consistency. Building upon this, we further integrate Feature Pyramid Network (FPN) layers, using 1 × 1 convolutions to dynamically fuse cross-scale features and establish inter-layer dependencies.

To illustrate the functional flow of the SPPELANF module in greater detail, let the input feature tensor be defined as:

(4) $$x\in R^{C_{in}\times H\times W}$$

To efficiently capture multi-scale contextual information under lightweight constraints, a 1 × 1 convolutional unit with batch normalization and LeakyReLU activation (referred to as CBS) is first applied to project the input from $c_{in}$ channels to an intermediate dimension $c_{*}$, yielding:

(5) y0=LeakyReluBNConv1×1x∈RC*×H×W

Subsequently, $y_{0}$ is passed through three successive max-pooling operations (with a kernel size of 5 × 5, stride of 1, and padding of 2), which progressively expand the receptive field while preserving the spatial dimensions.

(6) $$y_{1}=MaxPool\left(y_{0}\right),y_{2}=MaxPool\left(y_{1}\right),y_{3}=MaxPool\left(y_{2}\right)$$

where $y_{i}\in R^{C_{*}\times H\times W}$ for i=1,2,3. The resulting set $\left\{y_{0},y_{1},y_{2},y_{3}\right\}$ thus encodes hierarchical features ranging from fine-grained local patterns to broader global context.

To align these multi-scale features and ensure consistent channel dimensions, the following is defined:

(7) f0=y0,fi=LeakyReluBNConv1×1ix∈RC*×H×W,i=1,2,3

where each $Conv_{1\times 1}^{\left(i\right)}$ maps its input to $c_{*}$ output channels. $f_{0}$ directly reuses $y_{0}$ to avoid redundant computation. At this stage, all feature maps $\left\{f_{0},f_{1},f_{2},f_{3}\right\}$ share the same spatial resolution and number of channels.

These aligned features are concatenated along the channel dimension:

(8) $$Z=Concat\left(f_{0},f_{1},f_{2},f_{3}\right)\in R^{C_{*}\times H\times W}$$

**Figure 7:**
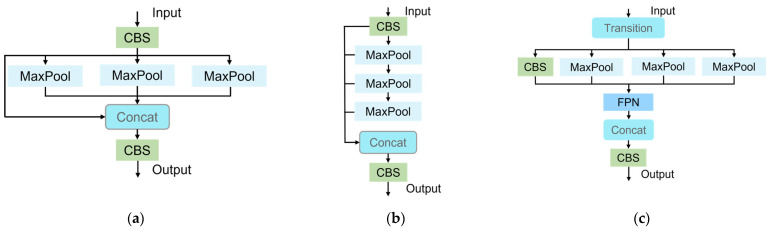
(**a**) SPP module structure, (**b**) SPPF module structure, (**c**) SPPELANF module structure.

This architectural design not only preserves fine-grained discriminative capability but also enhances adaptability to broiler face targets of varying scales. The inclusion of multi-scale pooling and lightweight FPN-style fusion effectively alleviates information loss due to occlusions commonly encountered in high-density farming environments, thereby improving the discriminative quality of broiler face representations in multi-object scenarios. Figure 7 provides an overall overview of the structures of the SPP, SPPF, and SPPELANF modules.

#### 2.3.6. CBAM Module

**Figure 8:**
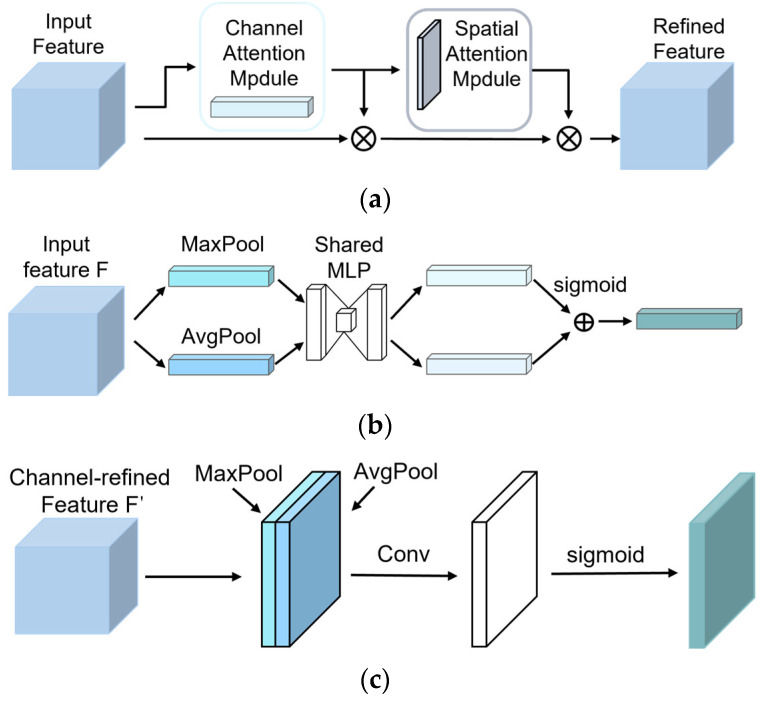
(**a**) CBAM module structure, (**b**) Channel Attention Module, (**c**) Spatial Attention Module.

As shown in Figure 8a, the CBAM (Convolutional Block Attention Module) enhances the network’s response to broiler face features by sequentially generating channel and spatial attention maps [36]. In the broiler face recognition task, the Channel Attention Module (CAM) strengthens the significant channel features related to broiler faces, improving the model’s sensitivity to these features. The Spatial Attention Module (SAM), on the other hand, optimizes attention to spatial positions, helping the network more accurately extract the details of the broiler face at different scales and spatial dimensions, particularly assisting in suppressing interference from complex backgrounds.

As shown in Figure 8b, CAM first generates spatial context descriptors FcAvg and FcMax through global max pooling and global average pooling, and then processes these inputs using a shared multi-layer perceptron (MLP) to obtain the channel attention map MC. This process allows the network to automatically focus on the key channel features in the broiler face image while ignoring background information. The computation formula for this process is:

(9) MC(F)=σ(MLP(AvgPool(F))+MLP(MaxPool(F)))

Then, MC is element-wise multiplied with the input feature map F, resulting in the broiler face feature map F′ adjusted by channel attention:

(10) $$F^{\prime }=MC(F)⊗F$$

As shown in Figure 8c, SAM generates spatial context features FsAvg and FsMax through global max pooling and global average pooling, respectively. These two features are concatenated, reduced in dimensionality using a 7 × 7 convolution, and passed through a Sigmoid activation to generate the spatial attention map MS. This process helps focus attention on the key spatial locations of the broiler face and suppress background interference. The computation formula is:

(11) $$MS(F^{\prime })=\sigma (f7\times 7([AvgPool(F^{\prime });MaxPool(F^{\prime })]))$$

Finally, MS is element-wise multiplied with F′, yielding the final spatial attention-adjusted feature map F″:

(12) $$F^{\prime \prime }=MS(F^{\prime })⊗F^{\prime }$$

Through these steps, CBAM effectively enhances the network’s feature representation ability across different scales and spatial dimensions, significantly improving the feature response in critical areas, particularly in broiler face recognition tasks.

### 2.4. Experimental Conditions and Parameter Settings

The experimental environment is configured with the Linux operating system, equipped with an NVIDIA GeForce RTX 3090 GPU (NVIDIA Corporation, Santa Clara, CA, USA), which has 24 GB of video memory. The deep learning framework used is PyTorch version 1.13.1, with the parallel computing platform set to CUDA 11.6, and the programming language environment is Python 3.8.0.

To optimize model training, a dynamic learning rate adjustment strategy is employed. This strategy includes two key parameters: the initial learning rate (lr0) and the learning rate decay factor (lrf). The initial learning rate serves as the baseline step size for model parameter updates, and its value directly affects the convergence speed in the early stages of training. A value that is too high may cause oscillation around the optimal solution, while a value that is too low will significantly extend the training period. The learning rate decay factor controls the magnitude of the learning rate decrease during training, and its value range is (0, 1). Specifically, the final learning rate is determined by the product of the initial learning rate and the decay factor. For example, when lr0 = 0.01 and lrf = 0.01, the learning rate at the end of training will drop to 0.0001. This progressive learning rate adjustment mechanism helps the model converge more stably.

In this experiment, a total of 500 epochs were set to ensure sufficient model training. The implementation of the dynamic learning rate strategy not only improves the stability of the training process, reducing fluctuations during parameter updates but also effectively facilitates convergence to the global optimal solution. Detailed hyperparameter configuration information is provided in Table 2.

### 2.5. Evaluation Metrics

To assess the performance of the improved recognition model, recall rate (R), average precision (AP), and mean average precision at a threshold of 0.5 (mAP@0.5) were used as evaluation metrics. The formulas for recall rate, AP, and mAP are as follows:

(13) $$AP=\int_{0}^{1}P\;dR$$

(14) $$mAP=\frac{1}{N}\sum_{i=1}^{N}AP_{i}$$

(15) $$Recall=\frac{TP}{TP+FN}$$

(16) $$Precision=\frac{TP}{TP+FP}$$

where TP represents true positives, FP represents false positives, FN represents false negatives, and TN represents true negatives. AP (Average Precision) is the area under the precision-recall (P-R) curve, used to measure the detection performance of a single class. N represents the number of classes, and mAP (mean Average Precision) is the average of AP values across all classes. A higher mAP value indicates better detection performance and recognition accuracy.

In addition to these performance metrics, this study also considers the model’s complexity, including the number of parameters and floating-point operations (FLOPs). For standard convolution, the formulas for calculating the number of parameters and FLOPs are as follows:

(17) $$Parameters=(K_{h}\times K_{w}\times C_{in})\times C_{out}+C_{out}$$

(18) $$FLOPs=[(K_{h}\times K_{w}\times C_{in})\times C_{out}+C_{out}]\times (H\times W)$$

where *K_h_* and *K_w_* represent the height and width of the convolution kernel, *C_in_* and *C_out_* represent the number of input and output channels, and *H* and W represent the height and width of the output feature map. Typically, *K_h_* and *K_w_* are of the same size.

## 3. Results

### 3.1. Quantitative Evaluation of Different Variants of the Inception-F Module in Multi-Scale Feature Extraction

To quantitatively evaluate the contribution of the five parallel paths (R1–R5) in the Inception-F module to multi-scale feature extraction, we first constructed three ablation variants: Inception-A (containing only R1–R3, as shown in Figure 5), Inception-B (containing R1–R4), and the complete Inception-F (containing R1–R5). These variants were used to replace the P3, P5, and P7 layers of the backbone network, respectively, and the experimental results are summarized in Table 3.

Experimental results demonstrate that Inception-F achieves significant improvements in precision, F1 score, and mean average precision (mAP) compared to the two ablation variants. Specifically, relative to the baseline model (YOLOv11n), Inception-F increases precision by 2.2 percentage points, F1 score by 0.8 percentage points, and mAP by 2.0 percentage points. Meanwhile, its parameter count and computational cost rise only marginally over those of Inception-A and Inception-B and remain substantially lower than the baseline. In contrast, the recall rates of Inception-A and Inception-B decrease by 1.3% and 0.9%, respectively, failing to balance overall performance. Considering both detection performance and model compactness, the full five-path dynamic weighted fusion variant (Inception-F) represents the optimal solution.

### 3.2. Comparative Experiments of the SPPELANF Module and Alternative Designs

To validate the effectiveness of the proposed parallel ELAN pooling with cross-layer FPN fusion strategy in the SPPELANF module, we compared it against two alternative configurations: the serial, same-scale max-pooling module (SPPF) from the baseline YOLOv11n and the parallel ELAN pooling module without FPN fusion (SPPELAN). Under the same training protocol, each design replaced the P9 layer of the backbone and was evaluated using identical performance metrics and complexity quantification methods. The results are summarized in Table 4.

Experimental results indicate that SPPELANF improves precision by 1.6 percentage points over the baseline and maintains near–optimal levels in F1 (87.2%) score and mAP (90.4%), while its parameter count and FLOPs differ minimally from those of SPPELAN. Although SPPELAN slightly outperforms SPPELANF in recall and mAP, its precision and F1 score are inferior, and the lack of cross-layer fusion leads to insufficient contextual consistency of features. In summary, the SPPELANF module achieves the optimal balance between detection performance and model compactness through parallel multi-scale extraction and cross-layer dynamic fusion.

### 3.3. Comparison of Inception-F Module Insertion Locations

To evaluate the impact of the Inception-F module on the performance of the YOLOv11n model, as shown in Figure 4, we inserted it into the P3, P5, and P7 layers of the backbone network (Position 1) and the P17 and P20 layers of the neck network (Position 2). Position 1 + 2 refers to the insertion at both positions. The results are shown in Table 5.

The experimental results show that the Inception-F module effectively improves mAP while reducing the model’s parameter size and FLOPs. At Position 1, the *p* value increased by 2.2%, and mAP improved by 2%. At Position 2, the *p* value decreased by 0.3%, but mAP increased by 1.6%. Although the *p* value at Position 1 + 2 decreased by 1%, its mAP significantly improved by 3.6%, reaching 91.4%. The parameter count for the Position 1 + 2 model decreased by 23.2%, and FLOPs were reduced by 16.6%. These results indicate that the Inception-F module not only improves mAP but also optimizes model lightweight design and computational efficiency. Despite fluctuations in *p* values at different positions, considering the significant mAP improvement and the notable lightweight effect, Position 1 + 2 was chosen as the final solution.

### 3.4. Comparison of C2f-Faster Module Insertion Locations

To evaluate the impact of the C2f-Faster module on the YOLOv11n model, we inserted it into the P2 and P4 layers of the backbone network (Position 1) and the P13 and P19 layers of the neck network (Position 2), and also considered inserting the module at both positions. The experimental results show that introducing the C2f-Faster module significantly reduced the model’s parameter count and FLOPs, while also improving mAP. The results are shown in Table 6.

Specifically, after inserting the module at Position 1, FLOPs decreased by 4.5%, mAP improved by 2.1%, and the parameter count reduced by 1.5%. At Position 2, FLOPs decreased by 7.5%, and mAP improved by 2.5%. Notably, when the C2f-Faster module was inserted at both Position 1 and Position 2, the reduction in FLOPs was the same as at Position 2, but the accuracy dropped by 1.5%. However, Position 2 maintained a high mAP (90.3%) and precision (90.5%), while significantly reducing computational load and memory usage. Considering the balance between model accuracy and computational resources, Position 2 was chosen as the optimal solution, especially for applications with limited computational resources.

### 3.5. Ablation Experiments

To systematically evaluate the impact of different modules on the performance of the YOLOv11n model, we designed several variant models and conducted ablation experiments. All experiments were conducted under the same dataset and training parameters to ensure that the comparison results accurately reflect the contribution of each improved module to object recognition performance. Additionally, we assessed the lightweight effects of each model to verify their feasibility on resource-limited embedded devices. As shown in Figure 4, YOLOv11-I represents the introduction of the Inception-F module, applied to the P3, P5, and P7 layers of the backbone network and the P17 and P20 layers of the neck network; YOLOv11-F represents the introduction of the C2f-Faster module, replacing the P13 and P19 layers of the neck network; YOLOv11-S represents the introduction of the SPPELANF module, replacing the SPPF module in the P9 layer of the backbone network; YOLOv11-C represents the introduction of the CBAM module, replacing the C2PSA module in the P10 layer of the backbone network. YOLOv11-IF combines the Inception-F and C2f-Faster modules, inheriting the improvements of YOLOv11-I and YOLOv11-F; YOLOv11-IFS further combines the SPPELANF module on this basis, inheriting the improvements of YOLOv11-I, YOLOv11-F, and YOLOv11-S; and the YOLO-IFSC model introduces all four modules (Inception-F, C2f-Faster, SPPELANF, CBAM) simultaneously, inheriting all the optimizations of YOLOv11-I, YOLOv11-F, YOLOv11-S, and YOLOv11-C. Through the comparison of these different variants, this study aims to explore the independent contributions of each module, the synergistic effects of module combinations, and their enhancements in model accuracy, recall rate, mAP, and lightweight design, particularly focusing on the adaptability and real-time processing capability for embedded devices. The results are shown in Table 7.

YOLOv11-I significantly improved the recall rate by 3.3%, and mAP increased by 3.6%, validating the effectiveness of the multi-scale feature fusion mechanism in complex scenarios. Although precision decreased by 1%, this indicates that a balance needs to be optimized between feature richness and classification confidence calibration. This performance improvement is attributed to the innovative design of the five parallel paths and dynamic weighting mechanism, which effectively extracts and integrates key features from multi-scale information. YOLOv11-F achieved a mAP of 90.3% with a 2.3% improvement in precision, indicating the advantages of PConv in enhancing local feature extraction. Although recall rate slightly decreased, the precision improvement effectively compensated for this gap. YOLOv11-S improved mAP by 2.6%, benefiting from the combination of Spatial Pyramid Pooling (SPP) and Feature Pyramid Networks (FPNs), which enhanced cross-scale information extraction and improved the processing accuracy for features at different scales. YOLOv11-C achieved a 1.7% improvement in precision, a 3.3% increase in recall rate, and a 2.4% improvement in F1 score. This improvement is attributed to the adaptive weighting in both the channel and spatial dimensions by the CBAM module, which effectively focuses on key features, enhancing the model’s recognition ability and accuracy. The YOLOv11-IF model demonstrated a significant lightweight advantage, with a 25.9% reduction in the number of parameters and a 19.6% reduction in FLOPs, while maintaining a mAP of 90.3%, proving that multi-module collaboration can achieve a balance between computational efficiency and recognition accuracy. Further integration of the SPPELANF module, forming YOLOv11-IFS, achieved a mAP of 91.2%, a 3.4% improvement over the baseline model, under the constraints of 1.79 M parameters and 5.2 G FLOPs. Finally, the integrated YOLO-IFSC model, with the cooperation of all four modules, achieved revolutionary lightweight design while maintaining excellent recognition performance (mAP50 = 91.5%, F1 = 87.3%): the number of parameters decreased to 1.55 M (a 40.8% reduction from the baseline), and FLOPs dropped to 5.0 G (a 24.2% reduction). The synergistic effect of all modules significantly enhanced the model’s comprehensive adaptability to complex scenarios, including multi-target interactions, dynamic scale variations, and robustness against environmental interference.

**Figure 9:**
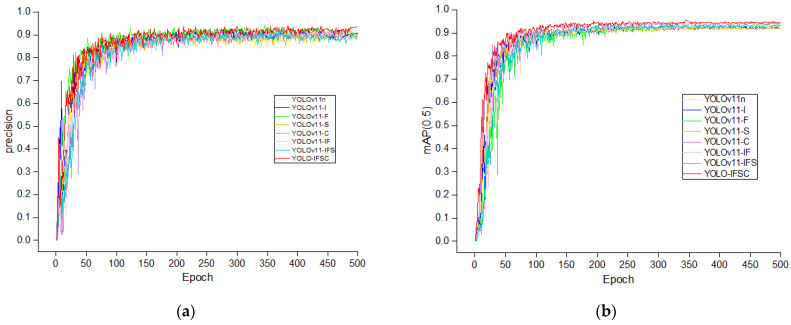
(**a**) Precision curve of all models during training process in ablation experiment, (**b**) mAP(0.5) curve of all models during training process in ablation experiment.

Figure 9 shows the precision and mAP (mean Average Precision) curves for the ablation experiments on the validation set. According to the experimental results, YOLO-IFSC not only achieves the highest mAP value, but also converges rapidly after only 100 epochs in the early stages of training, significantly outperforming other variants. In terms of precision, all models exhibit high smoothness and minimal fluctuation throughout the 500-epoch training process. This indicates that the synergistic effect of the modules significantly contributes to the improvement of recognition performance, particularly in mAP, and also validates the stability of the training process, providing a solid theoretical foundation and technical support for real-time object recognition tasks.

### 3.6. YOLO-IFSC Network Training Results

**Figure 10:**
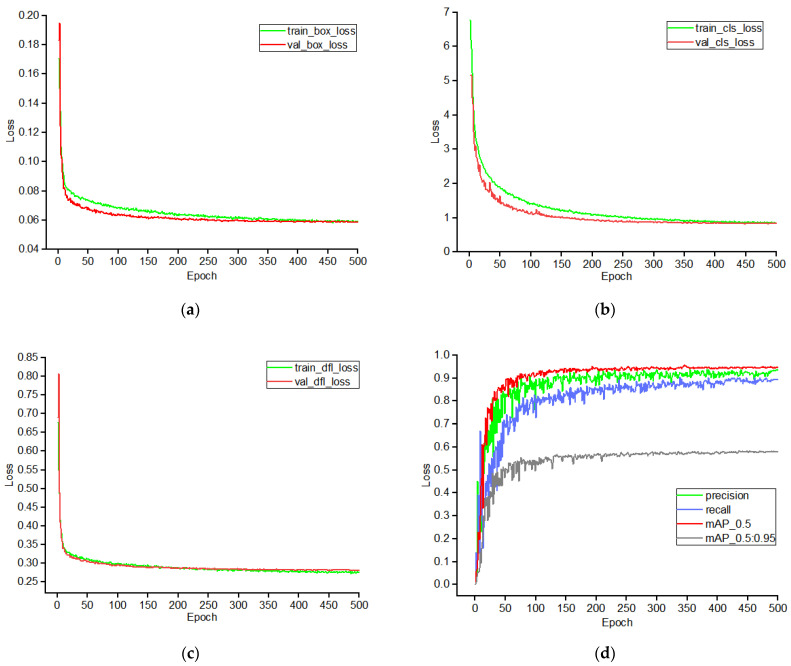
(**a**) Training loss and validation loss curve for box_loss during model training process, (**b**) training loss and validation loss curve for cls_loss during model training process, (**c**) training loss and validation loss curve for dfl_loss during model training process, (**d**) precision, recall, and mAP change curves during model training.

As shown in Figure 10, during the 500 epochs of training, both the training loss and validation loss showed a synchronized optimization trend: they rapidly decreased within the first 100 epochs, decelerated between 100 and 300 epochs, and stabilized from 300 to 500 epochs. The box_loss, cls_loss, and dfl_loss on the validation set all exhibited high stability, indicating that the model did not overfit during training and has good generalization ability. The changes in precision, recall rate, and mAP@0.5 followed the expected growth pattern, with mAP@0.5 stabilizing after about 100 epochs. Although recall rate showed some fluctuation in the early stages, overall metrics continued to improve, with no abnormal fluctuations or performance degradation, fully verifying the stability and reliability of the model’s recognition performance.

### 3.7. Progressive Occlusion Experiment

To quantitatively assess the model’s robustness under varying occlusion conditions, we manually synthesized three occlusion scenarios within the annotated broiler face bounding boxes using Photoshop’s rectangle tool: Level 1 (mild occlusion, covering ≤10% of the bounding box area and restricted to the periocular region); Level 2 (moderate occlusion, covering 20% ± 3% of the bounding box area, with the rectangular region spanning the eyes and lateral cheek regions and allowed to extend horizontally beyond the bounding box to realistically simulate large external occluders such as feeders or cage bars); and Level 3 (severe occlusion, covering 50% ± 5% of the bounding box area, encompassing the central and peripheral facial regions and permitting up to 5% of the occluder to exceed the bounding box to mimic more extreme physical obstructions).

**Figure 11:**
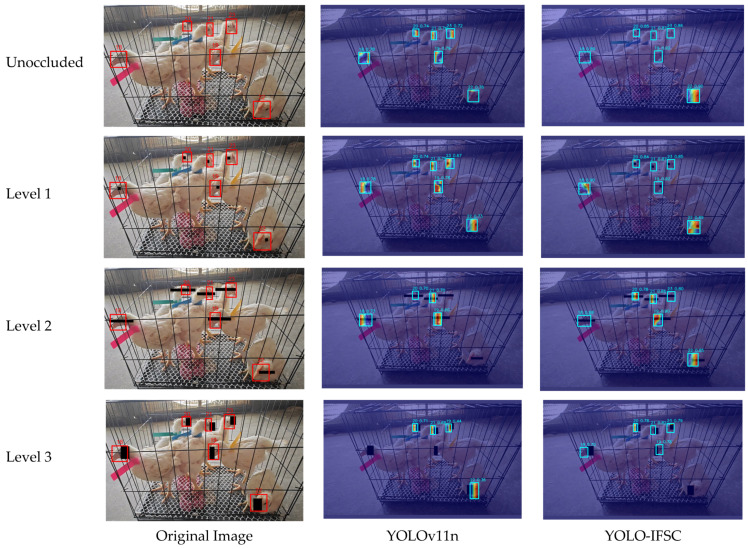
XGrad-CAM visualization of feature activation under different levels of synthetic occlusion (Level 1–3). Rows correspond to occlusion levels, and columns show results from YOLOv11n and YOLO-IFSC, respectively. Notably, YOLOv11n exhibited detection failures for Chicken 22 and 23 at Level 2 and 18 and 19 at Level 3, while YOLO-IFSC missed only Chicken 22 at Level 3.

The XGrad-CAM visualization results are shown in Figure 11. Compared to YOLOv11n, YOLO-IFSC exhibited more concentrated feature activation in the broiler face region. Quantitative analysis further verified the superior performance of YOLO-IFSC: at Level 1 and Level 2, zero-error recognition was achieved, and high confidence scores were maintained across all occlusion levels. In contrast, YOLOv11n showed identity recognition errors at Level 2, demonstrating lower robustness. These performance improvements were achieved without affecting real-time recognition efficiency (36.6 FPS), validating the effectiveness of the proposed architecture’s synergistic effects—multi-scale dynamic fusion, partial convolution kernel optimization, cross-layer feature integration, and dual-domain attention mechanism—in enhancing occlusion robustness and accuracy, making it suitable for broiler face recognition tasks.

### 3.8. Comparison with Different Models

To rigorously benchmark YOLO-IFSC on broiler face recognition, we compared it against four families of state-of-the-art detectors. First, a set of lightweight single-stage models—YOLOv3-tiny, YOLOv5s, YOLOv8n, YOLOv9s, YOLOv10n, and YOLOv11s—were chosen for their minimal parameter count and high inference throughput. Second, classic two-stage frameworks—Faster R-CNN and Cascade R-CNN—were included to represent architectures that prioritize robust feature extraction and detection accuracy. Third, transformer-based detectors—namely DETR and RT-DETR—were evaluated for their use of global self-attention in end-to-end set prediction and incorporation of deformable attention mechanisms to accelerate inference. Finally, RTMDet-tiny was assessed as a hybrid convolution–transformer design, fusing efficient convolutional blocks with attention mechanisms. The comparative results are presented in Table 8. This comprehensive evaluation highlights YOLO-IFSC’s superior trade-off between compactness and performance, underscoring its suitability for deployment in resource-constrained, real-time poultry-monitoring applications.

From the perspective of detection accuracy, YOLO-IFSC achieved an outstanding mAP@0.5 of 91.5%, significantly outperforming all lightweight models (e.g., YOLOv8n at 88.4% and YOLOv10n at 88.0%), representing improvements of 3.1% and 3.5%, respectively; compared to two-stage detectors Cascade R-CNN (89.4%) and Faster R-CNN (85.9%), YOLO-IFSC maintains high precision while incurring lower computational and storage overhead (only 1.55 M parameters and 5.0 GFLOPs), demonstrating a superior balance between model compactness and performance. Among transformer-based methods, DETR and RT-DETR achieve mAPs of 87.7% and 87.3% but require 41 M and 19.9 M parameters and 96 GFLOPs and 57 GFLOPs of computation, respectively—substantially higher than YOLO-IFSC, and thus less suitable for embedded deployment. Notably, RTMDet-tiny, as a hybrid lightweight–transformer architecture, attains a mAP@0.5 of 90.5%, approaching YOLO-IFSC’s accuracy, but exhibits inferior efficiency with an inference speed of only 29.1 FPS (versus YOLO-IFSC’s 36.6 FPS) and 4.88 M parameters with 8.0 GFLOPs—both exceeding those of our model and indicating higher deployment barriers. Overall, YOLO-IFSC delivers state-of-the-art detection accuracy and real-time performance under extremely low resource consumption, fully validating its applicability and promotion potential in resource-constrained environments.

### 3.9. Model Performance Evaluation on Embedded Platform

To assess the real-world deployment performance of the proposed YOLO-IFSC model on a resource-constrained embedded platform, this study utilized the NVIDIA Jetson Orin NX Super Developer Kit (NVIDIA Corporation, Santa Clara, CA, USA), which is equipped with an 8-core Arm Cortex-A78AE v8.2 CPU, a 1024-core Ampere GPU, and 16 GB of LPDDR5 memory. During testing, the model continuously received input data at a rate of 30 FPS. The system recorded key runtime metrics in real time, including end-to-end inference latency, peak GPU utilization, power consumption, and peak memory increase. The mean Average Precision (mAP) was subsequently computed offline to comprehensively evaluate the model’s detection accuracy and real-time responsiveness. The experimental results are summarized in Table 9.

YOLO-IFSC and its FP16 quantized version demonstrated superior performance over the baseline model YOLOv11n in terms of inference speed, resource consumption, and detection accuracy. Specifically, YOLO-IFSC achieved a high detection accuracy (mAP_50_ of 91.5%) with an end-to-end inference latency of 27.5 ms, while maintaining a peak GPU utilization of 71%, power consumption of 12.1 W, and memory increase of 1198 MB. After quantization, YOLO-IFSC_FP16 further reduced the inference latency to 17.4 ms, with power consumption and GPU utilization decreased to 10.7 W and 60%, respectively, and memory usage reduced to 933 MB, while detection accuracy only slightly dropped to 91.3%. In contrast, YOLOv11n and its FP16 variant exhibited inferior performance in terms of accuracy, inference speed, and resource efficiency. Additionally, the peak temperature remained well below the Jetson Orin NX’s thermal design limit of 85 °C, ensuring thermal stability and sustained high-performance operation during extended runtime. These findings demonstrate YOLO-IFSC’s exceptional balance of high accuracy, real-time responsiveness, and minimal resource demands, affirming its strong suitability for embedded deployment.

## 4. Discussion

Precision farming has raised an urgent demand for individual broiler recognition, and broiler face recognition, as a potential solution, plays a significant role in ensuring animal welfare and improving intelligent farming management. However, research on broiler face recognition is still in its early stages. This study proposes for the first time a lightweight, non-contact broiler face recognition model—YOLO-IFSC.

Ablation studies indicate that the strategy adopted in this study is both rational and superior. Firstly, large convolution kernels (e.g., the 5 × 5 convolution in Inception-F) expand the network’s receptive field, facilitating the capture of broader contextual information and enabling more complete modeling of shape features [49]. For example, models such as RepLKNet employ large kernels like 31 × 31 to construct an expanded receptive field [45], significantly enhancing object shape representation. Secondly, the introduction of partial convolution (PConv) helps eliminate redundant computations by performing depthwise convolution only on regions of interest. In broiler leg disease detection work, incorporating PConv into the C2f module not only reduced computational load but also more accurately extracted spatial features [34], which is consistent with our conclusions when applying this strategy in broiler face recognition. The inclusion of CBAM is also authoritatively supported: Woo et al. demonstrated that CBAM, as a lightweight module, can significantly improve the classification and detection performance of various CNNs [36]. Overall, the above strategies have been validated as effective through ablation experiments, and multiple studies have supported their design rationale, fully demonstrating the rationality and superiority of the methods employed in this work. Comparative experiments with other lightweight models further substantiate that our model achieves superior inference speed and accuracy. We additionally conducted model deployment experiments on the Jetson Orin NX platform to verify the application potential of YOLO-IFSC in real-world edge computing environments. The results showed that, under FP16 quantization, the model achieved an end-to-end inference latency of 17.4 ms with power consumption controlled below 11 W, demonstrating favorable real-time performance and energy efficiency. This performance level is comparable to existing lightweight detection models (such as YOLOv8n_FP16) on the same platform [50], and the model still maintained 91.3% mAP under embedded conditions, indicating that the proposed architecture not only ensures accuracy but also exhibits strong deployment adaptability and engineering feasibility.

However, there are certain limitations in this study. First, the current dataset primarily covers WOD168 broilers, and there is an urgent need to verify the model’s cross-breed generalization ability using multi-breed data. Second, as shown in Figure 11, in severe occlusion (Level 3) scenarios, some broiler identities were not accurately recognized, reflecting that there is still room for improvement in the model’s global dependency modeling. Moreover, variations in the growth stages of broiler chickens lead to facial feature drift, and the current model still requires improvement in its adaptability to different age groups. To address this, future work will focus on three directions: one is to introduce the long-range attention mechanism of the Vision Transformer [51] to enhance the model’s ability to capture contextual information in occluded areas; the second is to construct a diversified dataset covering multiple breeds to comprehensively improve the model’s generalization performance. Third, an online incremental learning strategy will be adopted to address feature drift across different growth stages. This study fills a critical technological gap in the niche domain of broiler facial recognition, representing a pivotal step toward individualized management in intensive farming environments. For the first time, YOLO-IFSC enables robust individual identification of broilers in high-density settings, laying a solid technical foundation for intelligent farm management, precision feeding, animal health and welfare monitoring, and full-process traceability from chick to market-ready product. Additionally, as the current dataset is still in collaboration with related enterprises and has not been made publicly available, it is planned to release the dataset after the project is completed to promote further research.

## 5. Conclusions

This study addresses the limitations of traditional RFID technology in broiler individual recognition and the inherent challenges of broiler face recognition, proposing a lightweight broiler face recognition model, YOLO-IFSC. By systematically integrating dynamic multi-branch feature fusion (Inception-F), partial convolution optimization (C2f-Faster), cross-layer pyramid aggregation (SPPELANF), and dual-domain attention mechanism (CBAM), the model significantly reduces computational redundancy and parameter count while enhancing multi-scale feature representation capabilities. Experimental results show that this model achieved excellent performance on a self-constructed dataset: mAP@0.5 reached 91.5%, inference speed was 36.6 FPS, and the parameter count was only 1.55 M, with computational complexity reduced to 5.0 GFLOPs. At the same time, the framework maintained a zero-error recognition rate under Level 1 and Level 2 occlusion scenarios and eliminated physical intervention through non-contact recognition, effectively improving animal welfare in precision farming environments. Moreover, the limitations of the current study lie in the model’s adaptability to extreme occlusions and cross-breed generalization, which still need to be improved. Future work will integrate the global context modeling ability of Vision Transformers and construct a multi-breed dataset to enhance the model’s universality. This study not only provides an efficient and lightweight solution for broiler face recognition, but also presents a methodological framework that can be extended to individual recognition tasks for other livestock and poultry, laying a theoretical and technical foundation for promoting the transition of the livestock industry from extensive management to precision and sustainable farming.

## Figures and Tables

**Table 1:** Dataset statistics for training, validation, and test splits (pre- and post-augmentation).

Statistic	Test Set	Validation Set	Original Training Set	Augmented Training Set	Total Training Set	Pre-Augmentation Total	Post-Augmentation Total
Number of Images	108	216	515	241	756	839	1080
Number of Face Annotations	416	852	2164	761	2925	3432	4193

**Table 2:** Experimental parameter settings.

Hyperparameter Selection	Setting
Input Image Size	640 × 640
Initial Learning Rate	0.01
Applied Learning Rate Coefficient	0.01
Momentum	0.937
Weight Decay	0.0005
Batch Size	16
Epoch	500

**Table 3:** Performance comparison of different variants of Inception-F module.

Model	P (%)	R (%)	F1 (%)	mAP (%)	Parameters (M)	FLOPs (G)
YOLOv11n	88.2	83.2	85.4	87.8	2.62	6.6
Inception-A	89.8	81.9	85.1	89.2	2.09	5.3
Inception-B	89.0	82.3	85.1	89.3	2.10	5.4
Inception-F	**90.4**	**83.1**	**86.** **2**	**89.8**	**2.** **11**	**5.5**

**Table 4:** Comparative results for SPPELANF module.

Model	P (%)	R (%)	F1 (%)	mAP (%)	Parameters (M)	FLOPs (G)
YOLOv11n	88.2	83.2	85.4	87.8	2.62	6.6
SPPELAN	87.6	**86.8**	86.9	90.5	2.42	**6.2**
SPPELANF	**89.8**	85.4	**87.2**	**90.4**	**2.42**	**6.2**

**Table 5:** Comparison of results by embedding Inception-F module at different layers of the network.

Model	P (%)	R (%)	F1 (%)	mAP (%)	Parameters (M)	FLOPs (G)
YOLOv11n	88.2	83.2	85.4	87.8	2.62	6.6
Position 1	**90.4**	83.1	86.2	89.8	2.11	5.5
Position 2	87.9	83.7	85.2	89.4	2.49	6.3
Position 1 + 2	87.2	**86.5**	**86.6**	**91.4**	**2.01**	**5.5**

**Table 6:** Comparison of results by embedding C2f-Faster module at different layers of the network.

Model	P (%)	R (%)	F1 (%)	mAP (%)	Parameters (M)	FLOPs (G)
YOLOv11n	88.2	83.2	85.4	87.8	2.62	6.6
Position 1	89.4	83.8	86.1	89.9	2.58	6.3
Position 2	**90.5**	84	**86.7**	**90.3**	2.55	**6.1**
Position 1 + 2	86.7	**85**	85.5	90.6	**2.52**	6.1

**Table 7:** Ablation experiment results.

Model	P (%)	R (%)	F1 (%)	mAP (%)	Parameters (M)	FLOPs (G)
YOLOv11n	88.2	83.2	85.4	87.8	2.62	6.6
YOLOv11-I	87.2	**86.5**	86.6	91.4	2.01	5.5
YOLOv11-F	90.5	84	86.7	90.3	2.52	6.1
YOLOv11-S	89.8	85.4	87.2	90.4	2.42	6.2
YOLOv11-C	89.9	**86.5**	**87.8**	90.8	2.34	6.2
YOLOv11-IF	90.2	83.3	86.4	90.3	1.94	5.3
YOLOv11-IFS	90.0	84.5	86.9	91.2	1.79	5.2
YOLOv11-IFSC	**91.4**	84.1	87.3	**91.5**	**1.55**	**5.0**

**Table 8:** Comparison of performance across different model series.

Model	P (%)	R (%)	mAP (%)	Parameters (M)	FLOPs (G)	FPS
YOLOv3_tiny	82.9	78.1	85.1	9.53	14.4	39
YOLOv5s	86.0	79.9	86.3	7.82	18.8	36.2
YOLOv8n	86.2	83.7	**88.4**	3.0	8.1	35.3
YOLOv9s	89.0	79.2	88.2	7.3	27.6	34.1
YOLOv10n	88.0	79.8	88.0	2.7	8.3	33.2
YOLOv11s	86.2	81.5	87.8	9.42	21.4	34.9
Faster_rcnn	82.7	78.3	85.9	41.39	208	31.9
Cascade_rcnn	85.1	83.3	89.4	69.29	236	25.6
DETR	86.2	**88.3**	87.7	41	96	39.8
RT-DETR	88.9	85.6	87.3	19.9	57	42.3
RTMDet_tiny	89.6	85.9	90.5	4.88	8	29.1
YOLO-IFSC	**91.4**	84.1	**91.5**	**1.55**	**5.0**	**36.6**

**Table 9:** Performance comparison of four models during inference on Jetson Orin NX.

Model	Peak Memory Increase (MB)	Peak Power Consumption (W)	Peak GPU Utilization (%)	Peak Temperature (°C, GPU/SoC)	End-to-End Latency (ms)	mAP (%)
YOLOv11n	1274	12.5	96	51.6/51.8	35.5	87.5
YOLOv11n_FP16	1061	10.7	70	51.3/51.7	22.6	87.6
YOLO-IFSC	1198	12.1	**71**	51.4/51.7	27.5	**91.5**
YOLO-IFSC_FP16	**933**	**10.7**	**60**	**50.9/51.3**	**17.4**	91.3

## Data Availability

The data provided in this study are available upon request from the corresponding author.
